# Glucan and Mannan—Two Peas in a Pod

**DOI:** 10.3390/ijms20133189

**Published:** 2019-06-29

**Authors:** Tatiana A. Korolenko, Nataliya P. Bgatova, Vaclav Vetvicka

**Affiliations:** 1 Department of Experimental Models of Neurodegeneration, Scientific Research Institute of Physiology and Basic Medicine, Timakov St. 4, 630117 Novosibirsk, Russia; 2 Laboratory of Electron Miscroscopy, Research Institute of Clinical and Experimental Lymphology—Affiliated Branch of Federal Research Center Institute of Cytology and Genetics, Siberian Division of the Russian Academy of Sciences, 630060 Novosibirsk, Russia; 3 Department of Pathology, University of Louisville, 511 S. Floyd, Louisville, KY 40292, USA

**Keywords:** polysaccharide, mannan, glucan, glucomannan, therapeutic, immunomodulator

## Abstract

In recent decades, various polysaccharides isolated from algae, mushrooms, yeast, and higher plants have attracted serious attention in the area of nutrition and medicine. The reasons include their low toxicity, rare negative side effects, relatively low price, and broad spectrum of therapeutic actions. The two most and best-studied polysaccharides are mannan and glucan. This review focused on their biological properties.

## 1. Introduction

The therapeutic potential of polysaccharides as regulators of macrophage activation is well established [1,2]. Carbohydrates are ubiquitous in all known organisms. The two most studied biologically active polysaccharides are mannan and glucan. The history of polysaccharides as immunomodulators goes back almost 80 years, to when Shear and Turner described a polysaccharide substance—again from *Serratia marcescens* cultures—that caused the necrosis of tumors [3]. Additional attention appeared when the first biological activity of zymosan was revealed. However, although a zymosan molecule can stimulate a nonspecific branch of immune response, it is not clear what component of this crude composition is responsible. According to chemical analyses, zymosan contains on average 55% of glucan, together with about 19% of mannan, 15% of protein, 6% of fat, and 3% of ash [4]. Pillemer et al. demonstrated that the essential activity of zymosan is connected with a glucan-rich fraction [5]. In contrast, the mannan-rich fraction, which is essentially a glucomannan protein, showed almost no activity [5]. These pioneering studies paved the way for the current boom in research on biological activities of polysaccharides. 

The cell wall of yeast is formed mostly of β-1,3-glucan, 1,6-glucan, mannan, and chitin. It is important to note that no obvious correlation could be found between β-glucan or mannan levels in yeast cultivated under different conditions [6]. With the increasing ability to isolate, characterize, and test cell wall-related polysaccharides, there is increasing commercial interest in the production of β-glucan and mannan for agriculture, pharmaceutical, and cosmetic purposes [7,8].

In addition to the action of individual mannans or glucans, glucomannan conjugates have been gaining significant attention lately. Besides their nutritional values, these conjugates have important health benefits such as the reduction of cholesterol and improvement of immune reactions [9,10]. 

In general, polysaccharides with immunostimulatory properties interact either indirectly or directly with various parts of the immune system and subsequently stimulate various immunological mechanisms. The main targets are cells of the monocyte lineage, but various other cell types, including T and B lymphocytes and fibroblasts, are also targets [2].

Despite longstanding research efforts, no clear conclusion has been made between the structure and function relationships (for review, see Ferreira, et al. [11]).

## 2. Mannans: Biological Function and Role in Pathology

The natural occurrence of mannans, their classification, structural differences, and significance in food and feed industries has been reviewed [12,13]. Generally, mannan is the generic name for the polysaccharide moiety of glycoproteins [14]. Mannan is usually a linear polymer of linked mannose residues [15]. The term “mannan” may also refer to a cell wall polysaccharide in yeasts. Yeast mannan contains an α(1-6)-linked backbone and α(1-2)-linked and α(1-3)-linked branches [12]. For details on mannan structure, see Moreira and Filho [12].

Mannan and the protein content of mannoproteins from yeast and the hyphal form of *Candida albicans* considerably differ [16]. Three major components of the yeast cell wall are mannan, glucan, and chitin, together forming approximately 90% of the entire cell wall. The ratio of these components significantly differs based on strain, environment, and growth stage [14]. The average percentage of mannan varies between 16–22% [17].

### 2.1. The Mannose Receptor

The mannose receptor (CD206) of macrophages is a C-type lectin, which has been shown to be expressed by macrophages, dendritic cells, and endothelial cells [18]. CD206 plays an important role in scavenging of mannoglycoproteins via endocytosis (phagocytosis) [19]. 

The mannose receptor is a transmembrane glycoprotein that is mainly expressed on the membrane of macrophages. It is specific for mannosylated molecules and subsequently mediates their endocytosis. In addition, evidence strongly suggests that the mannose receptor participates in the pathogen clearance [20], as it binds to mannose-containing and fucose-containing microorganisms via carbohydrate recognition domains. The binding and subsequent stimulation of phagocytosis by the macrophage mannose receptor actively triggers a release of secretory products such as IL-1, TNF-α, and reactive oxygen intermediates, further enhancing the antigen clearance [21].

Cells of the reticuloendothelial system recognize and bind mannose/N-acetylglucosamine (GlcNAC)-terminal glycoproteins, including some lysosomal enzymes [22]. In general, the mannose receptor is a highly effective endocytic receptor with a broad binding specificity [23]. Ligand specificity and cellular distribution give the mannose receptor a very important function in homeostasis and immune responses. This receptor serves as a homeostatic receptor by scavenging unwanted high mannose *N*-linked glycoproteins from the circulation. Since many pathogenic microbes are coated with mannose-containing structures, the mannose receptor interacts with those pathogens [18]. The mannose receptor functions in endocytosis and phagocytosis, and is deeply involved in immune homeostasis by scavenging unwanted mannoglycoproteins [18]. Mannan-binding lectin (mannose-binding lectin) participates in the innate immune system and is produced mainly by hepatocytes [24,25].

Mannose receptors are involved in the regulation of inflammatory responses, as they regulate the levels of molecules released into the circulation during inflammation. Therefore, mannose receptors are expressed at low levels during inflammation and at high levels at the end of inflammation, in order to ensure that the inflammatory agents are removed from circulation [26]. In addition, they play a role in switching M1 macrophage activation [27].

Expression of the mannose receptor has recently been demonstrated in the brain. Astrocytes and microglia are two types of glial cells that can convert to immunocompetent cells and represent the main sites of expression of this receptor *in vivo* and *in vitro*. The mannose receptor mediates in vitro pinocytosis by astrocytes and microglia and phagocytosis by microglia. Expression and endocytic activities of the mannose receptor in these cells are regulated by various cytokines [23].

It is important to note that this receptor recognizes a wide variety of microorganisms, and is often used by adapted intracellular pathogens. *Mycobacterium tuberculosis* can serve as an example, but other bacteria (such as *Cryptococcus*, *Streptococcus*, or *Yersinia*), viruses, and parasites can be similarly recognized [18]. This characteristic has been employed lately in the search for the optimization of CD206-mediated uptake by macrophages in various tissues during the development of infection. Most of the strategies involve the mannosylation of the carriers. This receptor serves as the recognition molecule of the lectin pathway of the activation of complement, as it is homologous to the C1q component. It also binds in a Ca-dependent manner to terminal mannose and GlcNAc residues. It also binds some glycoforms of IgG, IgA, and IgM, assisting in the clearance of immune complexes from blood [28].

Among the other pattern recognition receptors, the mannose receptor is among the best-characterized C-type lectins. It binds mannosyl, fucosyl, or N-acetylglucosamine (GlcNAc)-glycoconjugate ligands on various bacteria, fungi, and parasites to mediate significant inflammatory responses [25]. The mannose receptor also helps to clear glycosylated endogenous ligands. 

Dectin-1, another C-type lectin, is one of the major receptors for glucans. In this case, ligand binding potentiates phagocytosis and triggers reactive oxygen species (ROS) production in macrophages. Dectin-1 can cooperate with Toll-like receptor 2, thereby enhancing immune activation [29].

Other C-type–like receptors include DC-SIGN, DEC-205, and BDCA-2, which are mostly expressed on dendritic cells. These receptors participate in intertissue trafficking and communication and in ligand uptake, helping dendritic cells in their role of antigen-presenting cells. In addition to Kupffer cells and peritoneal and pulmonary macrophages, the activity of mannose receptors has been found in hepatic sinusoidal endothelial cells. Macrophages are primary targets for these vectors because of their importance in various diseases and resistance to gene transfer [30].

### 2.2. Mannans and Lysosomes

The therapeutic potential of carbohydrates is linked with their function as regulators of macrophage activation and is related to lysosomes [30]. Wattiaux et al. were the first to show that mannan (as well as yeast invertase: a glycoprotein reach in mannose) causes an in vivo increase (at a high dose, 10 mg injected into a rat, 15 h after) in the density of liver lysosomes (originating mainly from nonparenchymal cells, in which it accumulates, and to a lesser degree from hepatocytes) [31]. Using traditional methods of isopycnic centrifugation in a sucrose gradient and a distribution of several reference enzymes, those authors revealed a shift in the distribution of lysosomal enzymes arylsulfatase and cathepsin C to higher-density fractions of liver macrophages (and partially hepatocytes) without changes in marker enzymes in mitochondria and peroxisomes. This effect of mannan on liver lysosomes was found to be dose-dependent and is a result of difficulties with mannan hydrolysis in lysosomes. 

### 2.3. Hypolipidemic Effects of Mannan

Suppression of the proinflammatory activity of macrophages (M1 lineage) of the liver has a protective effect against a number of liver lesions (e.g., necrosis or fatty degeneration), and the mechanism remains unclear. Mannans as immunomodulators have been reported to stimulate macrophages in vivo via interaction with the mannose receptor [32]. Thus, they can participate in involving macrophages to eliminate circulating atherogenic lipoproteins [33,34].

Both adaptive and innate immune responses participate in every phase of atherosclerosis, thus tightly regulating the latter [35]. The immunomodulatory features of polysaccharides were employed to develop new approaches for the prevention and treatment of hyperlipidemia and atherosclerosis. Water-insoluble zymosan (a yeast cell wall polysaccharide) was shown to decrease the serum concentration of atherogenic lipids in experimental lipemia induced both by poloxamer 407 or Triton WR 1339 [36,37]. Lipopolysaccharides augment the uptake of oxidized low-density lipoprotein (LDL) by upregulating lectin-like oxidized LDL receptor 1 in macrophages [38]. So far, the hypolipidemic effects of mannan have not been studied in full detail. Mannans from *C. albicans* serotypes A and B have a comb-like structure consisting of two regions: one is acid-stable, and the second is acid-labile [32].

Mannan is a molecule supporting the effective elimination of circulating lipoproteins [34,39]. In our study, the mannan of *C. albicans* serotype A was used in a dose of 50 mg/kg (5×) or 100 mg/kg (2×) before acute lipemia was induced by the single administration of poloxamer 407 (250–300 mg/kg). In vitro treatment with mannan (50 μg/mL) stimulated the proliferative potential (*p* < 0.05) and nitric oxide (NO) production (*p* < 0.05) by peritoneal macrophages, which was comparable to the effects of β-glucan. Application of mannan A in mice with acute lipemia significantly (*p* < 0.001) reduced levels of the atherogenic LDL fraction as well as levels of total cholesterol and triglycerides (50 mg/kg dose produced better results) [34]. In liver, the total level of triglycerides decreased significantly; in serum, chitotriosidase activity increased after mannan-induced macrophage stimulation [33,34]. 

An experimental murine model with hyperlipidemic ApoE*3-Leiden showed that mannan supplementation influenced gut microbiota, increased the excretion of fecal bile acid, and decreased levels of plasma cholesterol and atherosclerosis development [40]. Accordingly, indigestible mannan oligosaccharides suppress the onset of atherosclerosis development by lowering plasma cholesterol levels; however, these oligosaccharides do not alleviate high-fat diet-induced obesity and glucose intolerance in obese C57BL/6 mice [41]. At the same time, according to other results, prebiotic mannan oligosaccharides improved the hypoglycemic effects of metformin, and this phenomenon correlates with modulation of the gut microbiota [42]. In veterinary medicine, a hydrolyzed-mannan-rich and glucan-rich yeast fraction was proposed to improve the health of cattle [43]. 

### 2.4. Electron Microscopy Study

**Figure 1:**
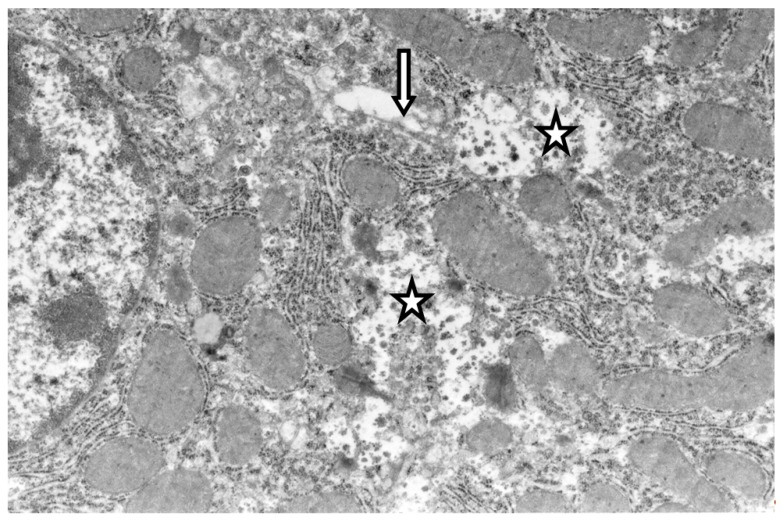
Fragment of hepatocyte cytoplasm of mice liver, 24 h after the single mannan A administration (50 mg/kg). Enlarged cisterns of the Golgi complex (arrow); decreased number of rosette of glycogen (stars). Magnification 6000×. All the experimental procedures were carried out in accordance with the guidelines of the NIH Guide for the Care and Use of Laboratory Animals and were approved by the Institutional Animal Care and Laboratory Animal Use Committee of the Scientific Research Institute of Physiology and Basic Medicine. This study was conducted in accordance with the recommendation of European Communities Council Directive of 22 September 2010 (2010/63/EU; Ethic Committee of the Scientific Research Institute of Physiology and Basic Medicine). All the experimental protocols were approved (2/05/2019) by the Ethic Committee of the Scientific Research Institute of Physiology and Basic Medicine. Confirmation of Biomedical Ethic Committee of State Institute of Physiology and Basic Medicine of Siberian Branch of Russian Academy of Medical Sciences, Novosibirsk, Russia Protocol № 5 from 11 September 2014 (Korolenko T.A. experimental work in mice with mannans).

**Figure 2:**
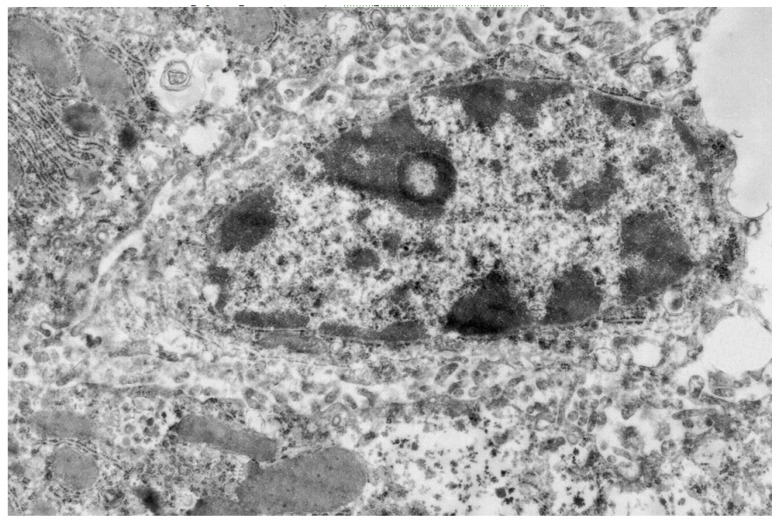
Liver macrophage fragment, 24 h after mannan A (50 mg/kg) administration. Magnification 6000×.

Evaluation of the hypolipidemic effects of mannans from *C. albicans* serotype A (mannan A) and serotype B (mannan B) in a murine model of hyperlipidemia has revealed that both mannans under study significantly stimulated both the proliferation and the NO production of murine peritoneal macrophages in vitro. Pretreatment of CBA/Lac mice with mannan A before the induction of hyperlipidemia significantly (*p* < 0.001) reduces serum levels of atherogenic LDL cholesterol, total cholesterol, and triglycerides [34]. However, according to the serum lipid profile, the mannan A hypolipidemic effect was weaker compared to atorvastatin [44]. Mannan A treatment induced the enlargement of complex Golgi cisterns and decreased the number of glycogen in hepatocytes (Figure 1). The ultrastructure of granular endoplasmic reticulum and number of free polysomal complexes were not changed (Figure 1); the liver macrophage did not reveal significant changes in ultrastructure (Figure 2).

**Figure 3:**
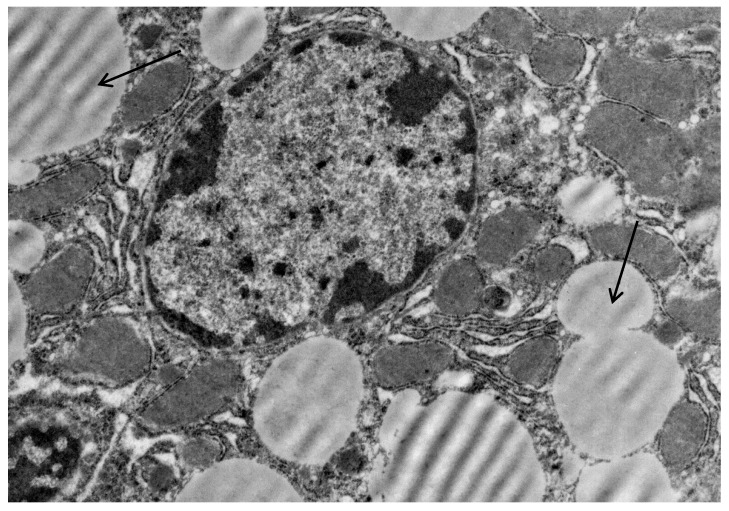
Liver cells of mice with acute lipemia induced by P-407 (250 mg/kg, 24 h). Fragment of hepatocyte of mice, 24 h after P-407 administration. The accumulation of lipid droplets (arrows). Magnification 6000×.

**Figure 4:**
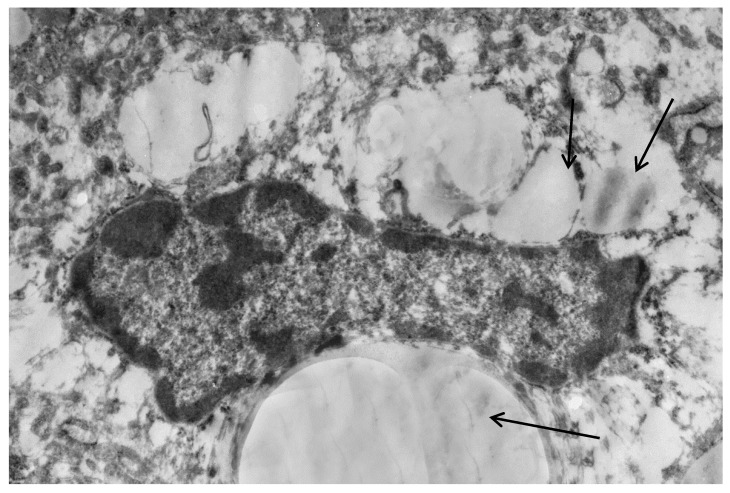
Liver macrophage in mice with acute lipemia, 24 h after P-407 administration. Lipids in cytoplasm (arrows). Magnification 6000×.

Poloxamer 407 administration in mice induced a significant accumulation of lipid droplets both in the cytoplasm of hepatocytes (Figure 3) and macrophages (Figure 4).

**Figure 5:**
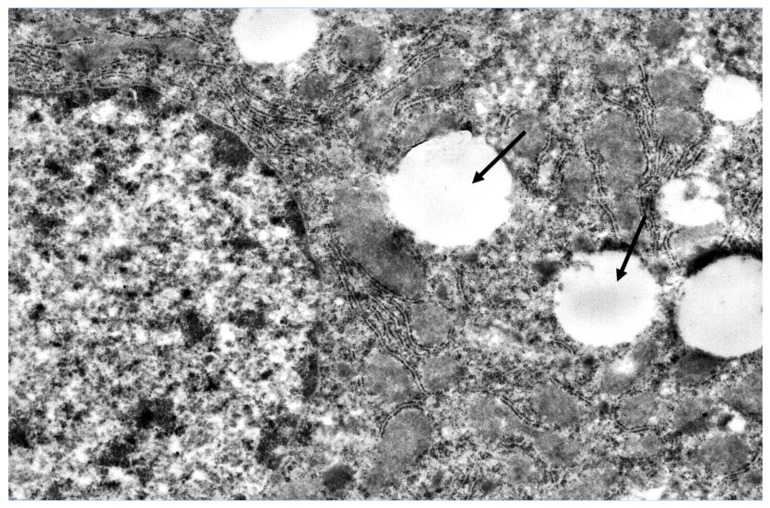
Mild lipid accumulation (arrows) in hepatocyte of mice with acute lipemia pretreated by mannan A. Magnification 6000×.

**Figure 6:**
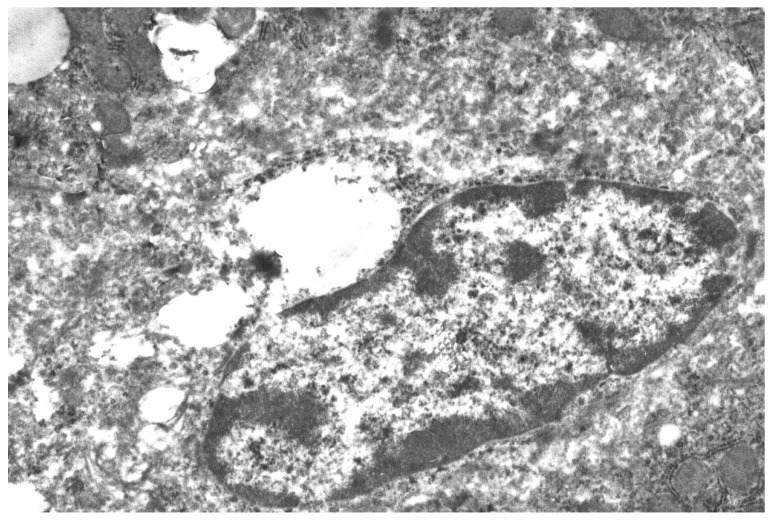
Liver macrophage in mice with acute lipemia pretreated by mannan A; decreased number of lipid droplets. Magnification 6000×.

**Figure 7:**
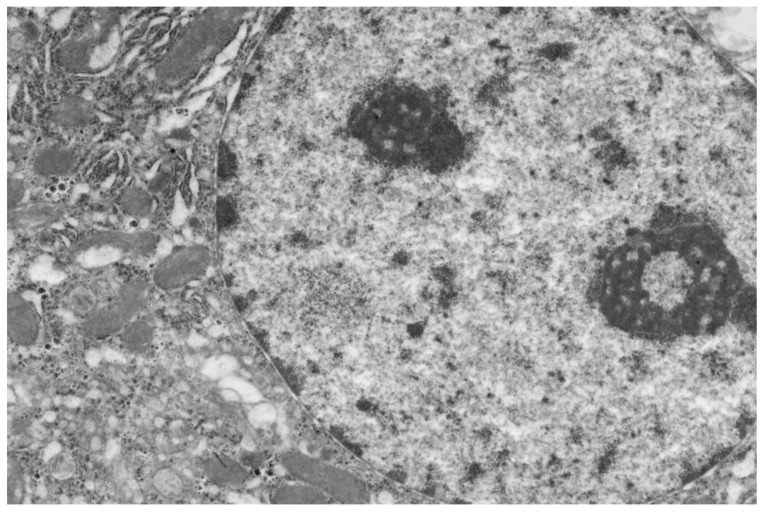
Effect of preliminary administration of mannan B in mice with acute lipemia induced by P-407 (250 mg/kg, 24 h). Fragment of hepatocytes: enlarged cisterns of Golgi complex and granular endoplasmic net; decreased amount of glycogen rosettes. Activation of nucleolar in hepatocyte nucleus. Magnification 6000×.

**Figure 8:**
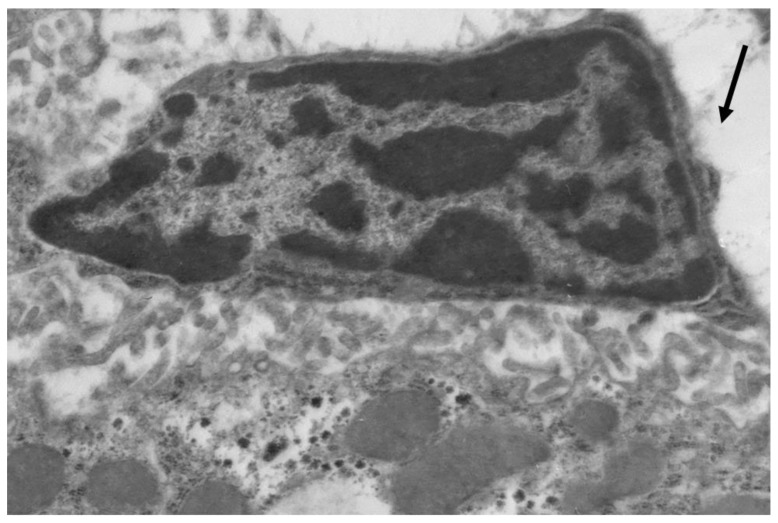
Lipid inclusions (arrow) in cytoplasm of macrophage in liver of mice 24 h after P-407-induced hyperlipidemia, effect of mannan B pretreatment. Magnification 6000×.

Mannan B exerts a more potent hypolipidemic effect than mannan A. Electron microscopic analysis of the liver revealed a strong reduction of number and volume of lipid droplets (Figure 5, Figure 6, Figure 7 and Figure 8). Thus, our findings suggest that both mannans in question may serve as effective lipid-lowering material, which could be used as a supplemental therapy to conventional hypolipidemic medication such as a statin treatment [33]. The situation in mice pretreated with mannan A is shown in Figure 5 and Figure 6.

In mice with acute lipemia pretreated by mannan B, we noted a decreased amount of lipid droplets, an enlargement of the cistern of the Golgi complex and granular endoplasmic reticulum, a decreased number of glycogen rosettes, and nucleolar activation in the nucleus of hepatocytes (see Figure 7 and Figure 8). The amount of accumulated lipid droplets decreased in mice with acute lipemia pretreated by mannan B (versus mice with P-407-induced lipemia) (Figure 8).

One can conclude that the results obtained so far point to a significant liver-protective activity of mannan and support more intensive research effort into possible application as a hypolipidemic molecule. Thus, mannan might be a future promising hypolipidemic drug similar to β-glucan.

### 2.5. Effects of Mannans in Tumors

Polysaccharides such as β-glucans and mannans have been reported to have antitumor and antimetastatic effects in lab experiments [36,45,46]. It was found that a brief treatment of lymphocytes with galactomannan (a galectin antagonist) prevents the binding to glycosylated receptors present on the membrane of tumor-infiltrating lymphocytes, reduces their motility on the cell surface, and corrects the functions of these cells [47]. Zhang et al. suggested a mannan-modified adenovirus encoding vascular endothelial growth factor receptor 2 (VEGFR2) as a vaccine for inducing antitumor immunity [48]. 

Tumor-associated macrophages perform essential functions: these cells mediate tumor angiogenesis, metastasis, and immune evasion [49]. Recently, a conjugate of alendronate-glucomannan (which is known to deplete tumor-associated macrophages) was suggested for cancer immunotherapy [50]. In both in vitro and in vivo assays, the conjugate preferentially accumulated in macrophages, thereby inducing apoptosis. Those authors have demonstrated that the delivery of therapeutic agents to eliminate tumor-associated macrophages can be a promising strategy for cancer immunotherapy. 

#### New Effects of Mannan That Are Related to Inflammation

There are some controversial data concerning the involvement of mannans in inflammation. On one hand, the beneficial antioxidant activities of mannan from the yeast cell wall were recently reported [51]. On the other hand, a single intraperitoneal injection of mannan from *Saccharomyces cerevisiae* was found to induce ROS-regulated, IL-17A-dependent psoriasis arthritis-like disease in mice [52]. This finding is suggestive of the possibility of new mechanisms that are triggered by exogenous microbial components, and subsequently result in the induction and exacerbation of psoriasis and psoriasis arthritis (IL-17 production, a pathway regulated by ROS in macrophages). The role of ROS, which were originally seen as toxic agents that will promote inflammation, is currently more oriented toward a hypothesis suggesting that ROS are crucial regulators of both immune and inflammatory pathways. Therefore, consistent with this assumption, mannose receptors are involved in protection against a possible pathogenic inflammatory macrophage response [53]. 

Additional studies found that compared to glucan or zymosan, mannan has only limited inflammatory effects in the pulmonary inflammation model [17]. Mannan treatment during allergen exposure had no activity as inflammation regulator [54]. An investigation of the protective effects in vomitoxin-induced oxidative stress suggested positive mannan effects, but the quality of the study was diminished by using rather crude mannan/glucan material with only 50.7% mannan, making a direct decision in which the molecule is really responsible for these effects rather difficult [55]. An indirect observation focused on the effects of obesity and metabolic homeostasis. These problems are known to be at least partly caused by inflammation. Mannan supplementation resulted in some immunomodulatory effects, but did not influence glucose intolerance or obesity, making the claims about the anti-inflammatory effects of mannan questionable [40,41].

According to a review by Singh et al., the low toxicity of mannans allows for their use in pharmaceutical, biomedical, cosmetics, and textile industries [13]. In the food industry, veterinary mannans have various applications such as gel formation, edible films/coating, stiffeners, viscosity modifiers, stabilizers, texture improvers, water absorbents, and as prebiotics in dairy products [56,57,58].

### 2.6. Mannan-Derived Oligosaccharides

Recently, significant attention was focused also on mannan-derived oligosaccharides (MOS), which have been used in numerous commercial products. The preparation is usually easy: yeast cells are lysed, and the resulting material is centrifuged. MOS supplementation was found to offer significant improvements of immune reactions and infection resistance in sea bass (*Dicenttrarchus labrax*), suggesting possible alternative to antibiotics [59]. In addition to fish, MOS, usually isolated from *S. cerevisiae*, were found to improve the average daily feed intake in pigs [60], chicken [61], crustaceans [62], and dairy calves [63,64]. The effects are usually manifested via the ability of MOS to influence the microbial population in the gastrointestinal tract, therefore serving as a probiotic [65].

## 3. β-Glucan

Detailed studies of the dynamics of the cell wall structure of *S. cerevisiae* revealed that glucans are primarily responsible for the mechanical strength of the cell wall. As the glucan chains are part of the hollow helix family, they are responsible for the elasticity of the cell wall [66].

Glucans are d-glucopyranosyl-based polysaccharides, which—based on their monosaccharide residues structure—can be α-d-glucans, β-d-glucans or α,β-d-glucans [67]. β-Glucans are the most studied polysaccharides. Similar to mannan, β-glucans are conserved structural components of the yeast, fungi, and seaweed cell walls. They are effective biological response modifiers that are able to both nonspecifically and specifically enhance the immune system [54]. After several decades of intensive research on biological effects, the properties β-glucan have been well established. Various studies have definitively shown that β-glucans represent important molecules with significant immunomodulatory functions [68] that have the ability to act through an organism’s own biological mechanisms as biological response modifiers [69].

These represent an evolutionary highly conserved structure that are often named PAMPs (pathogen-associated molecular patterns) (for review, see Zipfel and Robatzek [70]). Di Luzio et al., using the clearance of colloidal carbon, was the first to evaluate the effects of glucan on the phagocytic activity of macrophages [71]. These studies found that the mannan component of the yeast cell was biologically inactive (which was later found not to be entirely true), and the activity was still present in the zymosan molecule after the removal of lipids. Later, the major biological activity was found to be β-glucan.

More than 50 years ago, β-glucans were first described as biological response modifiers that could potentiate tumor rejection. At that time, they were classified as “nonspecific” because their molecular targets and the mechanisms of action were unknown, and their effects seemed to be pleiotropic and unpredictable. However, there is extensive literature about the activity of β-glucans in animal tumor models and, for the past 35 years, Japan has used several forms of mushroom-derived β-glucan, particularly lentinan and schizophyllan, in cancer patients. Recently, they are mostly used in conjunction with several types of traditional chemotherapy such as paclitaxel and cisplatin [72]. The main targets are macrophages, dendritic cells, monocytes, neutrophils, natural killer cells, and T lymphocytes.

Research with β-glucan has shown that it functions via the stimulation of granulocytes, dendritic cells, macrophages, lymphocytes, natural killer cells, and fibroblasts [68]. Two main β-glucan receptors have already been characterized on a molecular level. The first β-glucan receptor to be found and characterized was the iC3b-receptor known as CR3 [73,74]; the second was the Dectin-1 receptor [75]. In addition, receptors such as lactosylceramine (CDw17), langerin (CD207), and the family of scavenger receptors are known to be involved in β-glucan binding, but they are less studied and probably less important.

The innate immunostimulatory activities of β-glucan microparticles of baker´s yeasts origin were studied on mice experimental models [76,77,78]. Daily oral doses of 0.1 mg/kg of microparticulate β-glucan for two weeks significantly increased the phagocytic activity of peritoneal macrophages. β-Glucan microparticles applied in vitro enhanced T-cell activation and proliferation. In addition, other studies have demonstrated the enhanced phagocytose of β-glucan microparticles by peritoneal macrophages that was followed by the secretion of the proinflammatory cytokines (TNF-α, IL-6, and IL-1β) [79,80]. There are similar results documenting the stimulation of natural immunity factors in rat pulmonary macrophages [81] and in human mononuclear cells [82]. The study by Huang et al. confirmed increased TNF-α production in human dendritic cells primed by IFN-γ, which may decrease the response threshold of competent cells after stimulation by β-glucan microparticles [83]. 

The original studies on the effects that β-glucan has on the immune system focused on mice. Subsequent studies demonstrated that glucan can stimulate immune reactions in a wide variety of other species including earthworms, bees, shrimps, fish, chicken, rats, rabbits, guinea pigs, dogs, sheep, goats, pigs, cattle, horses, monkeys, and humans. Therefore, β-glucan can be considered an evolutionary old stimulant of many defense reactions [84]. Detailed reviews on β-glucan history and function are available [68,85,86,87].

β-Glucan has been used in clinical practice primarily in Japan, where it is used in cancer therapy since 1980 [88]. Western medicine was, and unfortunately still is, more reluctant. However, over 100 different clinical trials testing glucan supplementation in the treatment of various diseases are currently running all around the world. In our laboratory, we evaluated the effects of short-term glucan supplementation on the health conditions of children with chronic respiratory problems. In randomized, double-blind, placebo-driven studies, we compared the placebo group with a group supplemented with 100 mg of yeast-derived β-glucan for 30 days, and found significant improvements in the production of all classes of secretory immunoglobulins, and a higher secretion production of lysozyme, eNO, and calprotectin. In addition, strong improvements in endurance were observed [89,90,91]. Data from our clinical trials clearly supported our hypothesis that even short-time oral supplementation with β-glucan might have a positive effect on human health. 

Among less studied biological effects of β-glucans is the amelioration of stress-related problems, a reduction of the toxic effects of mercury, improvement in wound healing, the inhibition of atopic dermatitis, the reduction of periodontal bone loss, and an increase in the size and weight of fish larvae [92,93,94,95,96,97].

Besides these well-established biological effects of β-glucans, a completely new function was recently found. Newer and safer vaccine subunits such as proteins, peptides, or nucleic acids are being tested. However, their reduced immunogenicity has demanded the use of potent substances that could strengthen the immune response, principally working as adjuvants. Antigen encapsulation in polymer-based particles is a primordial tool for superior vaccine delivery to mucosal sites. The testing of suitable substances for these purposes is currently underway. Among these are particulate nanocarriers, which may exert a high adjuvant potential and could increase the immune response to vaccination due to their size and structural similarity by natural pathogens. These preparations are particularly advantageous for the nasal delivery of vaccines, which rapidly have become the favored vaccines because of the efficient M-cell uptake in the nasal-associated lymphoid tissue. Cevher et al. described the various compositions of glucan-based materials for nasal deliveries [98].

From a series of biopolymers, β-glucans seem to be the most promising. β-Glucans in the form of microparticles could serve not only as immunostimulants but also as antigen carriers. They could be advantageously applied to mucosal vaccination [99]. As a particular system, β-glucan particles are especially attractive as carriers, since they feature the targeted delivery of antigens and offer inherent adjuvant function. The addition of antibodies to this mix further improved the specific targeting [100].

In addition, the baker’s yeast (*S. cerevisiae*)-derived glucan microparticles could be regarded as promising for an oral delivery platform [101]. Yeast are characterized by high glucan content (more than 85% β-1,3-d-glucan polymers) with a small admixture of chitin (about 2%) and lipids (less than 1%) [102]. Capsular yeast shell microparticles were used to deliver ovalbumin to dendritic cells. The particles are well recognized by dendritic cells, and upon internalization trigger the release of costimulatory molecules [103].

Based on our current knowledge, it seems that the low intrinsic immunogenicity, a lack of toxicity, high and well-documented immunomodulating properties, good biocompatibility, and cost-effectiveness in large-scale manufacturing make β-glucan a promising new candidate for novel vaccine design. More research is necessary to select the best adjuvant to challenge the monopoly of aluminum adjuvants in human and animal vaccines.

β-Glucan clearly provides pleiotropic effects on various aspects of biological reactions. Despite intensive research in the last three decades, our knowledge of the mechanism of functions is still not complete. However, it is universally accepted that the time when β-glucan will be introduced as a drug in Western medicine is getting closer.

## 4. Glucomannan Conjugates

Glucomannans are carbohydrate polymers with a linear polymeric sequence consisting of 1,4-β-d-mannose and 1,4,β-d-glucose residues. Some studies suggested a presence of galactose residues [104]. It usually exists in the form of a soluble, viscous fiber. Since a human amylase enzyme cannot split these linkages, glucomannans move to the colon, where they are fermented by various bacteria [105].

One of the most promising glucomannan conjugates in konjac, which is a polysaccharide of *Amorphophallus konjac*. This glucomannan has long been used in traditional medicine in Japan and China. The health benefits include a reduction of blood glucose, reduction of obesity, regulation of lipid metabolism, amelioration of inflammatory bowel disease, and anti-inflammatory activity (for a review, see Behera and Ray [106]). A detailed study evaluating the effects of an eight-week course of dietary supplements on fish found that C-reactive protein and IgM levels were significantly increased, regardless of the dose. Supplementation also increased IL-6 expression in the anterior gut, TNF-α expression in some gut segments, and IL-1β expression in the distal gut [107].

A *Candida utilis* glucomannan has the potential to treat exaggerated neutrophilic inflammation, thus offering a new treatment method for rheumatoid arthritis [108].

Most glucomannan effects are connected with its structure as a fiber. It promotes satiety via increased mastication effort [109]. High viscosity also helps to slow small bowel transit.

Clinical trials found that at doses around 3 g/day, glucomannans were well tolerated, and their consumption resulted in weight loss in obese and overweight volunteers. In addition, food supplementation with glucomannans improved glycemic status and lipoprotein parameters (for a review, see Keithley and Swanson [110]). However, some clinical studies offered contradictory results. A clinical trial evaluating the safety and efficacy of eight-week supplementation with 1.33 g/day of glucomannan found no effects on body weight or glucose and lipid parameters [111]. A meta-analysis of randomized trials of glucomannans revealed that glucomannans did not affect the body weight, plasma lipid levels, blood pressure, or fasting blood glucose [112].

Besides the possible effects of metabolism via their fibrous structure, glucomannans have been linked with other biological activities. Among these reactions is the stimulation of fibroblasts with the subsequent increase of production of collagen around wounds and burns [113]. Among other claims are some effects on infections, but the results are still conflicting, and the studies usually lacking significant impact (for a review, see Tester and Al-Ghazzewi [114]).

## 5. Summary

The applications and functions of these commonly used and commercially available mannans and glucans have been described in the literature. Mannans improve the texture and appeal of food products and offer numerous health benefits such as control of obesity and weight control in general, prebiotic benefits, constipation alleviation, the prevention of diarrhea, inhibition of inflammation due to gut-related diseases, management of diverticular disease, balancing of the intestinal microbiota, immune-system modulation, and risk reduction for colorectal cancer [10,21,36,56,57,58]. Mannan-degrading enzymes represent the key for mannan degradation and are useful in various industrial processes, such as fruit juice clarification and a reduction in the viscosity of coffee extracts, as well as facilitating some process stages and improving process quality [12].

On the other hand, the major biological effects of β-glucans are oriented toward the immune system. With over 20,000 scientific publications and approximately 80 ongoing clinical trials worldwide, β-glucan is getting closer to overcoming the “immunomodulator” label and being accepted as a drug. 

With biological activities of both polysaccharides established, the theory of even higher activities of their combination (i.e., glucomannan) was not approved. The reasoning may be that despite dozens if not hundreds of individual β-glucans differing in any possible physicochemical characteristics, only one biologically active glucomannan exists. 

The two polysaccharides described in this review are most probably the two best-studied polysaccharides with significant biological and most of all immunological activities.
